# Synaptophysin controls synaptobrevin-II retrieval *via* a cryptic C-terminal interaction site

**DOI:** 10.1016/j.jbc.2021.100266

**Published:** 2021-01-08

**Authors:** Callista B. Harper, Eva-Maria Blumrich, Michael A. Cousin

**Affiliations:** 1 Centre for Discovery Brain Sciences, University of Edinburgh, Edinburgh, Scotland, EH8 9XD, UK; 2 Muir Maxwell Epilepsy Centre, University of Edinburgh, Edinburgh, Scotland, EH8 9XD, UK; 3 Simons Initiative for the Developing Brain, University of Edinburgh, Edinburgh, Scotland, EH8 9XD, UK

**Keywords:** synapse, vesicles, endocytosis, exocytosis, neuron, neurotransmitter release, synaptosome, DIV, days *in vitro*, mCer, mCerulean, GST, glutathione-S-transferase, SV, synaptic vesicle, Syp, Synaptophysin, SybII, Synaptobrevin-II

## Abstract

The accurate retrieval of synaptic vesicle (SV) proteins during endocytosis is essential for the maintenance of neurotransmission. Synaptophysin (Syp) and synaptobrevin-II (SybII) are the most abundant proteins on SVs. Neurons lacking Syp display defects in the activity-dependent retrieval of SybII and a general slowing of SV endocytosis. To determine the role of the cytoplasmic C terminus of Syp in the control of these two events, we performed molecular replacement studies in primary cultures of Syp knockout neurons using genetically encoded reporters of SV cargo trafficking at physiological temperatures. Under these conditions, we discovered, 1) no slowing in SV endocytosis in Syp knockout neurons, and 2) a continued defect in SybII retrieval in knockout neurons expressing a form of Syp lacking its C terminus. Sequential truncations of the Syp C-terminus revealed a cryptic interaction site for the SNARE motif of SybII that was concealed in the full-length form. This suggests that a conformational change within the Syp C terminus is key to permitting SybII binding and thus its accurate retrieval. Furthermore, this study reveals that the sole presynaptic role of Syp is the control of SybII retrieval, since no defect in SV endocytosis kinetics was observed at physiological temperatures.

The correct formation of synaptic vesicles (SVs) by endocytosis after their activity-dependent fusion is essential for the maintenance of neurotransmission. To be functionally competent, SVs must be packaged with a specific complement of lipids and proteins in a defined stoichiometry (1, 2). Most SV proteins contain peptide motifs enabling clustering by adaptor protein complexes such as AP-2 (3). Furthermore, monomeric adaptor proteins facilitate the incorporation of specific SV proteins such as synaptobrevin-II (SybII) and synaptotagmin-1 respectively into SVs (4, 5). Finally, SV protein interactions themselves are important for efficient retrieval. In particular, synaptophysin (Syp) and SV2A facilitate the accurate trafficking of SybII and synaptotagmin-1 during SV endocytosis (6, 7, 8, 9). These proteins are termed intrinsic trafficking partners, and this cotrafficking may provide a molecular explanation for protein stoichiometry on SVs (10, 11).

Syp associates with SybII both *in vitro* and *in vivo* (12, 13, 14, 15, 16). They are proposed to interact *via* their transmembrane domains, since binding is retained on deletion of one or more of their cytoplasmic regions (16, 17, 18, 19). However, a definitive interaction site for either protein has not been identified.

Syp knockout neurons display impaired SybII retrieval from the plasma membrane (7, 20, 21, 22) and slowed SV endocytosis (7, 20, 23, 24). However, the molecular mechanism that underpins these defects remains unclear. The major potential protein–protein interaction interface on Syp is its cytoplasmic C terminus (approximately 90 amino acids), previously proposed to control SV endocytosis kinetics during stimulation (23). The C terminus is also implicated in SybII retrieval, since a disease-associated frame-shift mutation within the C terminus disrupts this process when expressed in Syp knockout neurons (20). We therefore set out to establish whether the Syp C terminus has distinct molecular roles in SybII retrieval and SV endocytosis kinetics.

We reveal that the only physiologically relevant role for Syp is the activity-dependent trafficking of SybII, with its cytoplasmic C terminus essential for this process. Furthermore, we discovered a cryptic interaction site for the SybII SNARE motif within the Syp C terminus, suggesting an intramolecular conformational change within Syp permits the SybII interaction.

## Results

### The Syp C terminus is essential for accurate sybII retrieval

**Figure 1:**
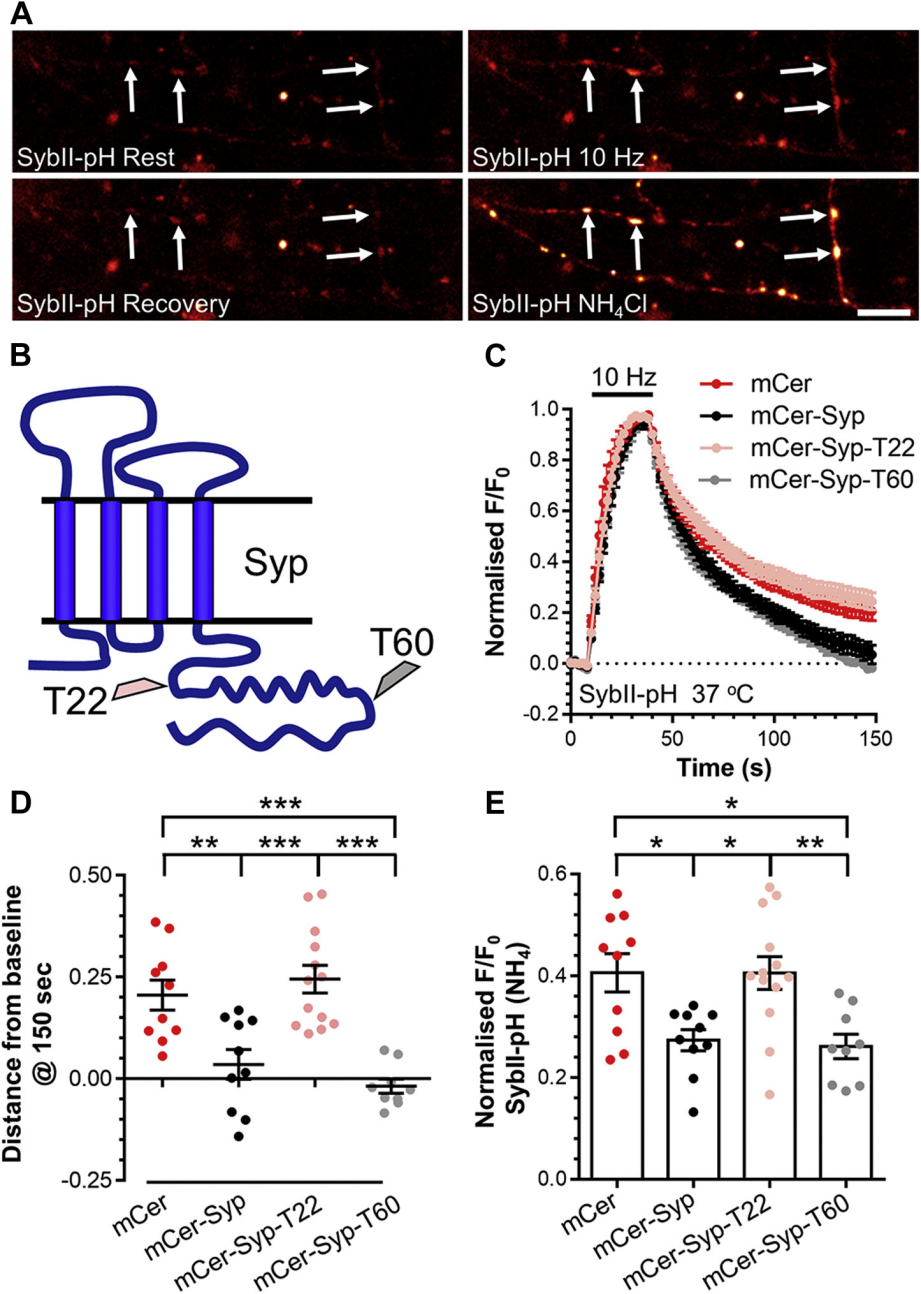
**The Syp C terminus is essential for accurate SybII retrieval.** Primary cultures of Syp knockout hippocampal neurons were transfected with SybII-pHluorin and mCer-Syp, mCer-Syp-T22, mCer-Syp-T60, or mCer between 7 and 8 DIV. At 13 to 16 DIV, at 37 °C, neurons were stimulated with action potentials (10 Hz, 30 s). Neurons were pulsed with NH_4_Cl imaging buffer after 180 s. *A*, representative images of neurons transfected with mCer-Syp and SybII-pH are displayed at Rest (t = 0 s), 10 Hz, Recovery (t = 150 s) and NH_4_Cl. *Arrows* indicate nerve terminals; scale bar = 10 μm. Truncations are displayed in *B*. *C*, average fluorescent SybII-pHluorin response (F/F_0_ ± SEM) normalized to the stimulation peak (indicated by bar, n = 10 mCer, mCer-Syp, n = 13 T22, n = 9 T60). *D*, SybII-pHluorin response at 150 s (F/F_0_ ± SEM). *E*, evoked SybII-pHluorin response normalized to the NH_4_Cl (F/F_0_ ± SEM). *D* and *E*, one-way ANOVA, all conditions compared with significant differences shown by ∗*p* < 0.05, ∗∗*p* < 0.01, ∗∗∗*p* < 0.001.

We examined SybII retrieval using a molecular replacement strategy in primary hippocampal cultures of Syp knockout neurons. Two Syp mutants were investigated, in addition to either wild-type Syp tagged with the fluorescent protein mCerulean (mCer-Syp) or the empty mCer vector. The first mutant was truncated at amino acid K242, retaining 22% of C-terminal amino acids (mCer-Syp-T22, Fig. 1*B*). This mutant is almost identical to one that failed to rescue SV endocytosis kinetics during stimulation in Syp knockout neurons (23). The second mutant was truncated at amino acid P276, retaining 60% of the C terminus (mCer-Syp-T60, Fig. 1*B*). This truncation is at the position of a disease-related frame-shift mutation in Syp, which rescued SV endocytosis kinetics but not SybII retrieval (20).

SybII retrieval was monitored using the genetically encoded reporter SybII-pHluorin, which indicates the pH of its immediate environment due to a pH-sensitive GFP (pHluorin) fused to its intraluminal C terminus (25). At rest, SybII-pHluorin fluorescence is quenched in the acidic SV lumen. During neuronal activity, arrival at the plasma membrane (and exposure to the neutral extracellular environment) is detected as an increase in fluorescence (Fig. 1*A*). Following stimulation, the kinetics of the fluorescence decay reflects the speed of SybII-pHluorin retrieval, since endocytosis is rate limiting when compared with SV acidification ((26, 27) but also see (28)).

Syp knockout neurons were cotransfected with SybII-pHluorin and mCer-Syp mutants, with SV recycling evoked *via* 300 action potentials delivered at 10 Hz. Experiments were performed at 37 °C, to ensure that any observed effects were physiologically relevant. Stimulation of Syp knockout neurons expressing wild-type mCer-Syp resulted in an increase in SybII-pHluorin fluorescence due to SV exocytosis, which returned to baseline after termination of the stimulus (7) (Fig. 1, *C* and *D*). In contrast, the SybII-pHluorin response failed to return to baseline in Syp knockout neurons (mCer), indicating impaired retrieval (7, 20) (Fig. 1, *C* and *D*). Furthermore, these neurons displayed a significantly larger evoked SybII-pHluorin peak, due to perturbed SybII retrieval during stimulation (23) (Fig. 1*E*). Expression of mCer-Syp-T22 failed to rescue the increase in evoked peak height (Fig. 1*E*), consistent with previous work (23). Surprisingly, this mutant also failed to rescue the poststimulation SybII-pHluorin response (Fig. 1*D*), suggesting a role for the Syp C-terminus in SybII retrieval both during and after neuronal activity. In contrast, expression of mCer-Syp-T60 fully rescued both the evoked peak height and retrieval kinetics of SybII-pHluorin (Fig. 1, *D* and *E*). Therefore, the Syp C terminus performs a key role in the activity-dependent trafficking of SybII with a region between K242 and P276 essential for this function.

**Figure 2:**
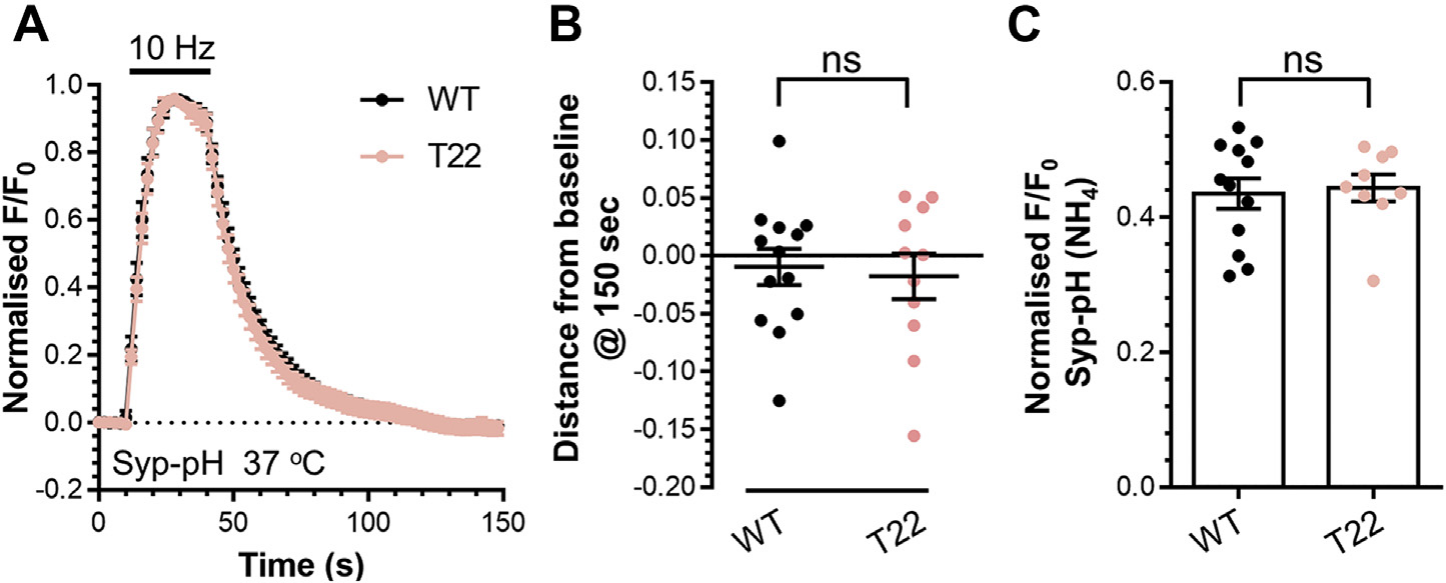
**The C terminus is dispensable for Syp trafficking.** Primary cultures of Syp knockout hippocampal neurons were transfected with either Syp-pHluorin (WT) or T22 Syp-pHluorin between 7 and 8 DIV. At 13 to 16 DIV, at 37 °C, neurons were stimulated with action potentials (10 Hz, 30 s). Neurons were pulsed with NH_4_Cl imaging buffer after 180 s. *A*, average fluorescent Syp-pHluorin response (F/F_0_ ± SEM) normalized to the stimulation peak (indicated by bar, n = 13 WT, n = 11 T22) (*B*) Syp-pHluorin response at 150 s (F/F_0_ ± SEM). *C*, evoked Syp-pHluorin response normalized to the NH_4_Cl (F/F_0_ ± SEM). Student’s *t*-test, *B*, *p* = 0.74, *C*, *p* = 0.63.

The failure of mCer-Syp-T22 to rescue SybII-pHluorin trafficking could be due to the truncation altering the trafficking of Syp. To address this, we examined the activity-dependent trafficking of Syp-pHluorin in Syp knockout neurons either with or without this truncation. These experiments revealed that Syp-pHluorin with a T22 truncation displayed identical trafficking to wild-type (Fig. 2). Therefore, the failure of the T22 truncation to rescue defects in the activity-dependent SybII trafficking was not due to altered Syp trafficking.

### The Syp C terminus is dispensable for SV endocytosis kinetics

**Figure 3:**
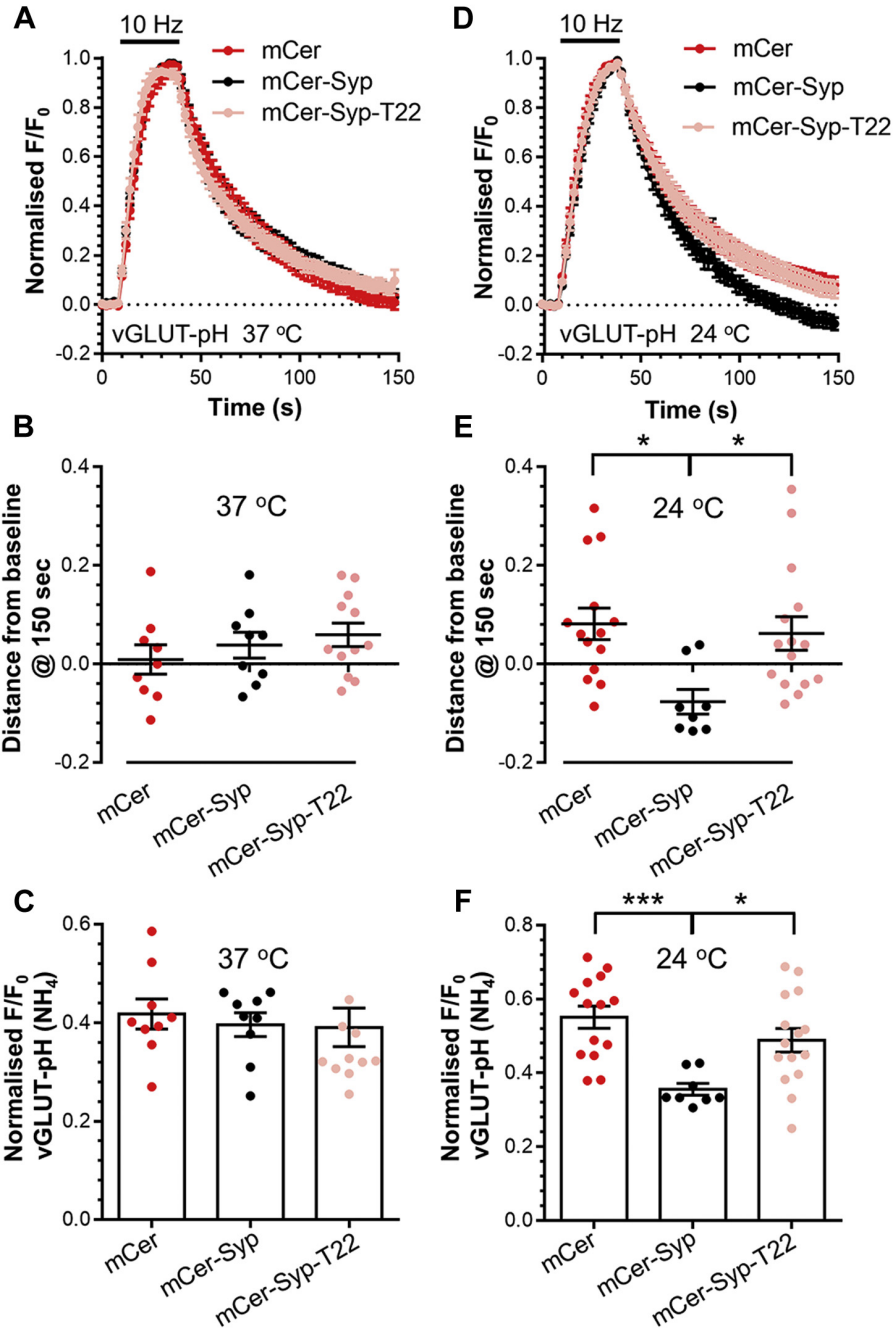
**Syp does not control endocytosis kinetics at 37 °C.** Primary cultures of Syp knockout hippocampal neurons were transfected with vGLUT-pHluorin and either mCer-Syp, mCer-Syp-T22, or mCer alone between 7 and 8 DIV. At 13 to 16 DIV, neurons were stimulated with action potentials (10 Hz, 30 s). Neurons were pulsed with NH_4_Cl imaging buffer after 180 s. Experiments were performed at either 37 °C (*A*–*C*), or 24 °C (*D*–*F*). *A* and *D*, average fluorescent vGLUT-pHluorin response (F/F_0_ ± SEM) normalized to the stimulation peak (indicated by bar, *A*; n = 9 mCer, mCer-Syp, n = 12 T22 *D*; n = 14 mCer, n = 8 mCer-Syp, n = 15 T22). *B* and *E*, vGLUT-pHluorin response at 150 s (F/F_0_ ± SEM). *C* and *F* evoked vGLUT-pHluorin response normalized to NH_4_Cl (F/F_0_ ± SEM). *B*, *C*, *E* and *F*, one-way ANOVA, all conditions compared with significant differences shown by ∗∗∗*p* < 0.001, ∗*p* < 0.05.

The fact that Syp-pHluorin-T22 displayed unaltered activity-dependent trafficking suggests that SV recycling is also unaffected by loss of the C terminus. To confirm this, we monitored SV recycling using the reporter, vGLUT-pHluorin (29), which was coexpressed with mCer, mCer-Syp, or mCer-Syp-T22. There was no difference in either the evoked peak height or the kinetics of vGLUT-pHluorin retrieval between Syp knockout neurons and those expressing either wild-type mCer-Syp or mCer-Syp-T22 (Fig. 3, *A*–*C*). Therefore, deletion of the Syp C terminus has no impact on SV recycling kinetics.

This result was surprising, since a slowing in SV endocytosis has been observed in Syp knockout neurons (7, 20, 23, 24). We reasoned that the absence of an effect might be a consequence of performing experiments at physiological temperature. We therefore repeated these experiments at room temperature. Under these conditions, a defect in both the evoked peak height and poststimulation recovery of vGLUT-pHluorin fluorescence was apparent in the absence of Syp (Fig. 3, *D*–*F*), even though the recovery kinetics were surprisingly faster at room temperature. Furthermore, mCer-Syp-T22 was unable to rescue either parameter (Fig. 3, *D*–*F*). Since these defects were absent at physiological temperatures, it suggests that the only role for Syp at central nerve terminals is the control of SybII retrieval during SV endocytosis.

### The Syp C terminus contains a cryptic interaction site for SybII

**Figure 4:**
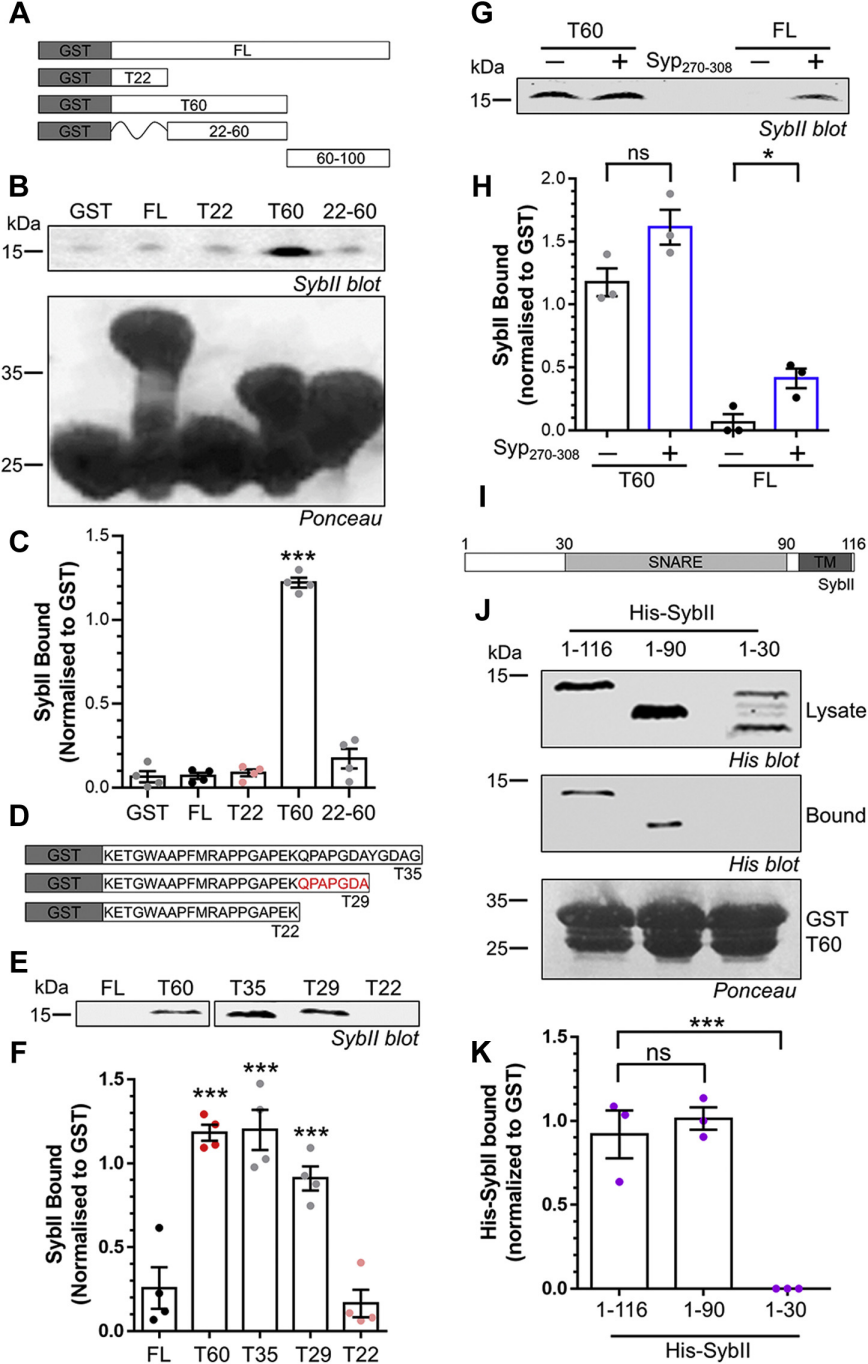
**The SypC terminus contains a cryptic SybII interaction site.***A* and *D*, Syp C-terminal GST-fusion proteins and Syp_270–308_ peptide. *B*–*F*, GST-fusion proteins were incubated with nerve terminal lysates and SybII binding determined by western blot. *B* and *E*, representative SybII blot and Ponceau stain (to reveal GST fusion proteins). *C* and *F*, quantification of SybII binding, normalized to GST fusion protein (±SEM, all n = 4, ∗∗∗*p* < 0.001 one-way ANOVA to GST). *G*, GST-Syp-C-FL or T60 were incubated with Syp_270–308_ peptide for 1 h, before washing and addition of nerve terminal lysate. Representative SybII blot is displayed. *H*, quantification of SybII binding, normalized to GST fusion protein (±SEM all n = 3, Student’s *t*-test, FL *p* = 0.07, T60 *p* = 0.026). *I*, SybII structure. *J*, GST-Syp-C-T60 was incubated with bacterial lysates expressing full-length His-SybII (1–116), 1 to 90 or 1 to 30 truncations. Representative His blots and Ponceau stain are displayed. *K*, quantification of His-SybII binding, normalized to His expression and GST fusion protein (±SEM, all n = 3, ∗∗∗*p* < 0.001 one-way ANOVA to FL).

The ability of mCer-Syp-T60, but not mCer-Syp-T22, to rescue activity-dependent SybII-pHluorin trafficking suggests that the region between T22 and T60 contains a SybII interaction site. Therefore, we determined whether the Syp C terminus with these truncations could bind to SybII. To achieve this, the Syp C terminus was fused to glutathione-S-transferase (GST) to generate affinity columns, which were then incubated with nerve terminal lysates (Fig. 4*A*). The extent of SybII binding was examined by western blotting. GST-Syp-C-T22 displayed no SybII binding over background GST levels (Fig. 4, *B* and *C*), as predicted from its inability to rescue SybII-pHluorin trafficking. In contrast, GST-Syp-C-T60 displayed strong binding to SybII (Fig. 4, *B* and *C*), in agreement with the rescue of SybII-pHluorin retrieval.

We next determined whether the region between the T22 and T60 truncations was sufficient to bind SybII by generating a fusion protein encompassing this sequence (GST-Syp-C-22-60). This fusion protein did not bind to SybII (Fig. 4, *B* and *C*), indicating that GST-Syp-C-T22 must contain part of the SybII interaction site. Surprisingly, full-length Syp C terminus (GST-Syp-C-FL) displayed no binding to SybII over background levels (Fig. 4, *B* and *C*). Therefore, SybII only interacts with Syp if the distal portion of the C terminus is removed.

Further truncation studies (Fig. 4*D*) revealed that removal of seven amino acids C terminal to T22 (QPAPGDA) was sufficient to ablate SybII binding, suggesting this region is essential for the interaction (Fig. 4, *E* and *F*). Therefore, a cryptic SybII interaction site resides within the first 26 amino acids of the Syp C terminus (residues 219–244). This site is occluded by the full sequence, suggesting sybII interactions are controlled by the distal Syp C-terminus. To test this, we synthesized a peptide identical to this distal region (Syp_270–308_) and examined its ability to modulate SybII binding to either GST-Syp-C-FL or GST-Syp-C-T60. In the absence of peptide, GST-Syp-C-T60 bound SybII while GST-Syp-C-FL did not, as observed previously (Fig. 4, *G* and *H*). In the presence of Syp_270–308_, SybII binding to GST-Syp-C-T60 was retained, suggesting it did not interfere with the interaction. Interestingly, Syp_270–308_ facilitated an interaction between GST-Syp-C-FL and SybII (Fig. 4, *G* and *H*), significantly increasing binding of SybII. This suggests that the distal region of Syp is key to revealing a cryptic SybII interaction site within the C terminus.

To determine the region of SybII that interacts with the Syp cryptic interaction site, sequential truncations of His-tagged SybII were performed and their ability to be extracted from bacterial lysates by GST-Syp-C-T60 was determined (Fig. 4*I*). Both full-length (residues 1–116) and the cytoplasmic domain (1–90) of His-SybII bound to GST-Syp-C-T60 (Fig. 4, *J* and *K*). However, deletion of the SybII SNARE motif (1, 2, 3, 4, 5, 6, 7, 8, 9, 10, 11, 12, 13, 14, 15, 16, 17, 18, 19, 20, 21, 22, 23, 24, 25, 26, 27, 28, 29, 30) resulted in a loss of binding (Fig. 4, *J* and *K*). Therefore, the SybII SNARE motif is the interaction interface for the cryptic Syp binding domain.

## Discussion

Syp is reported to control both the activity-dependent trafficking of SybII and SV endocytosis kinetics (7, 20, 23, 24). Here we reveal that the only physiologically relevant role for Syp in SV recycling is the control of SybII retrieval. In addition, we discovered a key role for the Syp C-terminus, with SybII retrieval controlled *via* a cryptic interaction site.

Two mutants were chosen for this study. The T22 mutant mimics a truncation that slowed SV endocytosis kinetics during stimulation in Syp knockout neurons (23), whereas the T60 is truncated at the site of a disease-associated frame-shift mutation that perturbed SybII retrieval (20). The full rescue of SybII-pHluorin trafficking by T60 suggests that the reported defects were due to the additional amino acids added after the frame shift.

Syp and SybII form a complex in nerve terminals (12, 13, 14). Subsequent work characterized how this complex was regulated by neuronal activity, development, intracellular calcium and the lipid microenvironment (15, 16, 30, 31, 32, 33, 34). In spite of this, a definitive explanation of how these SV proteins interact is still absent. Previous studies have hinted that they interact *via* their transmembrane domains, since removal of the Syp C terminus in either yeast two-hybrid assays (19) or a heterologous expression system (18) had small effects on binding. Here, we reveal a clear functional role for the C terminus in the retrieval of SybII during SV endocytosis. The interaction site is within the first 26 amino acids of the C terminus (219–244), with residues 238–244 being essential. The remainder of the C terminus is intrinsically disordered, consisting of multiple tyrosine-based pentapeptide repeats (35, 36). Recent studies examining a similarly disordered region of synapsin-1, demonstrated it could form a liquid phase at a sufficiently high concentration (37). This may explain why SybII is excluded from binding by the full-length Syp C terminus and why this interaction has not been previously observed. Tyrosine phosphorylation of the Syp C terminus does not appear to modulate SybII binding, since phospho-mimetic and null substitutions had no modulatory effect (data not shown).

The interaction with SybII was revealed *via* the addition of a peptide sequence corresponding to the final 40% of the Syp C terminus. This peptide may prevent the accretion of the Syp C terminus described above, permitting SybII binding. Alternatively, it may displace an independent interaction partner. The Syp C terminus interacts with AP-1 *via* its pentapeptide repeats (38) and Siah-1A/Siah-2 *via* its extreme C terminus (39). However, a key point to note is that the Syp_270–308_ peptide facilitates SybII binding when preincubated with the Syp C terminus and is then removed before addition of nerve terminal lysate. This strongly suggests that Syp_270–308_ is disrupting an intramolecular interaction within the C terminus, allowing SybII to bind.

We revealed that SybII interacts with Syp *via* its SNARE motif, with no contribution from its transmembrane domain. The C-terminal region of the SNARE motif may be essential for this, since recombinant SybII encompassing residues 68–116 extracts Syp from SV lysates (17). This is attractive, since the monomeric adaptor AP180 interacts with the N-terminal SNARE region to mediate SybII retrieval (40). Thus both Syp and AP180 may act in concert to facilitate SybII retrieval (10).

How could this interaction occur *in vivo*? The structure of Syp/SybII complexes immunoprecipitated from brain has been revealed using negative stain electron microscopy (41). In this structure, 12 copies of SybII intercalate between six Syp molecules in a rosette-like structure. Whether SybII enters a preassembled Syp rosette after SV fusion or whether this structure spontaneously assembles in the plasma membrane is still unclear. This structure may enable clustering of Syp and SybII molecules for retrieval in the correct stoichiometry to that observed on SVs (1, 2). Furthermore, Syp binding to the SybII SNARE motif may permit upstream binding by AP180 (10).

This study reveals that Syp has a single physiological role in SV recycling, the accurate trafficking, and retrieval of SybII. We propose that after SV fusion, the *cis*-SNARE complex is cleared from the active zone *via* an interaction between SybII and intersectin (42). The SNARE complex is broken apart through the action of NSF (43, 44), before SybII is captured by Syp (10). Syp restricts the entry of SybII into futile *cis*-SNARE complexes by interacting with its SNARE domain, while presenting it in the correct configuration for its retrieval by AP180 (4, 40).

## Experimental procedures

### Materials

Tissue culture reagents were from Invitrogen (Paisley, UK), except foetal bovine serum (Biosera, France) and papain (Worthington, USA). Nitrocellulose membranes and molecular weight markers were from BioRad (Perth, UK). Primary antibodies were from Abcam (Cambridge, UK) unless specified. All other reagents were from Sigma-Aldrich (Poole, UK).

Syp-pHluorin was from Prof. Leon Lagnado (University of Sussex), vGLUT-pHluorin from Prof. Robert Edwards (University of California), SybII-pHluorin from Prof. Gero Miesenboeck (University of Oxford), and mCer-Syp was generated as described (20). Truncations were generated using site-directed mutagenesis by adding a stop codon after amino acids K242 (T22) and P276 (T60). T22 truncated rat Syp-pHluorin was generated by adding a stop codon after K237. Mouse Syp C-terminus (residues 219–308) was ligated into a PGEX-KG vector (from Dr Colin Rickman, Heriot-Watt University) using XhoI and HindIII enzymes (forward primer CTCGAGTCAAGGAGACAGGCTGGGCCGCCCC; reverse primer AAGCTTTTACATCTGATTGGAGAAGGAGGTG (restriction sites underlined). The Syp C terminus was truncated by adding a stop codon after amino acids K237 (T22) A244 (T29), G249 (T35), and G268 (T60). GST-Syp-C-22-60 was generated using the forward primer TAAGCACTCGAGCAACCGGCACCCGGGGACGCCTACG and reverse primer TGCTTAAAGCTTAAGGCTGGTAGCCGCCCTGAGGCCC. Syp_270–308_ (mouse residues 270–308) was generated by BioServUK Ltd (Sheffield, UK). Mouse SybII (residues 1–116) was cloned into a pQE-30 vector (Quiagen, UK) using BamHI and SalI enzymes (forward primer GGATCCATGTCGGCTACCGCTGCCACCGTCC; reverse primer GTCGACCTAAGTGCTGAAGTAAACGATGATGATG. His-SybII (1–30) and (1–90) were generated by adding a stop codon after amino acids R30 and W90.

### Animal maintenance

All animal work was performed in accordance with the UK Animal (Scientific Procedures) Act 1986, under Project and Personal Licence authority approved by the University of Edinburgh Animal Welfare and Ethical Review Body (Home Office project licence—7008878). Animals were killed by schedule 1 procedures in accordance with UK Home Office Guidelines; adults were killed by cervical dislocation followed by exsanguination, embryos were killed by decapitation followed by destruction of the brain. Syp knockout mice (45) were maintained as heterozygotes on a C57BL/6J background and timed mated as homozygous pairs.

### Primary neuronal culture and transfection

Dissociated primary hippocampal-enriched neuronal cultures were prepared from E16.5 to 18.5 embryos from Syp knockout mice of both sexes (8, 21). Neurons were plated at 3 to 5 × 10^4^ cells on poly-D-lysine and laminin-coated 25 mm coverslips. Cells were transfected on 7 to 8 days *in vitro* (DIV) with Lipofectamine 2000 (20).

### Fluorescence imaging

Primary cultures were used at 13–16 DIV. Live fluorescence imaging was performed on a Zeiss Axio Observer D1 or Z1 inverted epifluorescence microscope (Cambridge, UK) with a Zeiss EC Plan Neofluar 40x/1.30 oil immersion objective. Cultures were mounted in an imaging chamber with embedded parallel platinum wires (RC-21BRFS, Warner Instruments, USA) and stimulated with 300 action potentials delivered at 10 Hz (100 mA, 1 ms pulse width). Imaging buffer (in mM: 119 NaCl, 2.5 KCl, 2 CaCl_2_, 2 MgCl_2_, 30 D-glucose, 25 HEPES, pH 7.4 supplemented with 10 μM 6-cyano-7-nitroquinoxaline-2,3-dione and 50 μM DL-2-Amino-5-phosphonopentanoic acid) was continuously perfused at either 37 °C or 24 °C (VC66-CS system, Warner Instruments, USA). After 180 s cultures were perfused with alkaline imaging buffer (50 mM NH_4_Cl substituted for 50 mM NaCl) to reveal total pHluorin fluorescence. Images were captured using an AxioCam 506 mono camera (Zeiss), with pHluorin or mCer vectors visualized at either 500 nm or 430 nm excitation (long-pass emission filter >520 nm). Each experimental condition was sampled on the same day, within the same set of primary cultures.

Offline data processing was performed using Fiji is just ImageJ software (46). A background thresholding script was used to select nerve terminals responding to stimulation. Average fluorescent intensity was measured using the Time Series Analyzer plugin. Subsequent data analyses were performed using Microsoft Excel, Matlab (Cambridge, UK) and GraphPad Prism 6.0 (CA, USA) software. The activity-dependent pHluorin fluorescence change was calculated as F/F_0_ and normalized to fluorescence at either the stimulation peak or in the presence of NH_4_Cl.

### Protein expression and GST pull-downs

Isolated nerve terminals were prepared from rat brains of both sexes (47). GST fusion proteins were expressed and coupled to glutathione-Sepharose beads (48). Nerve terminals were solubilized for 5 min at 4 °C in 25 mM Tris, pH 7.4, with 1% Triton X-100, 150 mM NaCl, 1 mM EGTA, 1 mM EDTA, 1 mM PMSF, and protease inhibitor cocktail. Bacteria expressing His-SybII proteins were lysed in 20 mM HEPES, 200 mM KCl, 50 mM imidazole, 2 mM β-mercaptoethanol, 10% v/v glycerol, 1% v/v Triton X-100, pH 7. Synaptosome or bacterial lysates were centrifuged at 20,442*g* for 5 min at 4 °C with the subsequent supernatant incubated with GST-fusion proteins for 1 h at 4 °C unless otherwise indicated. After washing in lysis buffer (including a 500 mM NaCl wash), beads were washed in 20 mM Tris (pH 7.4) and boiled in SDS sample buffer. The released proteins were separated by SDS-PAGE for western blotting analysis (anti-SybII, ab3347, 1:1000; anti-His, H1029, 1:3000). IRDye secondary antibodies (800CW anti-rabbit IgG, #925-32213, 1:10,000) and Odyssey blocking PBS buffer were from LI-COR Biosciences (Nebraska, USA). Blots were visualized using a LiCOR Odyssey fluorescent imaging system, with band densities quantified using either LiCOR Image Studio Lite software (version 5.2) or Image J (version 1.52). The SybII band was normalized to the GST fusion protein band revealed by Ponceau-S staining (His-SybII was also normalized to bacterial expression). Where indicated, Syp_270–308_ was incubated with GST fusion proteins for 1 h, before washing and addition of nerve terminal lysate.

### Statistical analysis

Statistical analysis was performed in Graph Pad Prism 6.0. Sample size (n) for neuronal cultures was individual coverslips and for synaptosomes, individual experiments. All data are presented as mean values ±standard error of the mean (SEM). For comparisons between two groups, a Student’s *t*-test was used, for more than two groups, a one-way ANOVA was performed with a post-hoc Tukey test when comparing all conditions and a Dunnett test when comparing to one condition (both corrected for multiple comparisons).

## Data availability

All relevant data are contained within the article.

## Conflicts of interest

The authors declare that they have no conflicts of interest with the contents of this article.
